# Supplementary material for: Content-rich biological network constructed by mining PubMed abstracts
Source: BMC Bioinformatics. 2004 Oct 8;5:147. doi: 10.1186/1471-2105-5-147 (PMC528731; doi:10.1186/1471-2105-5-147)
Supplement: Additional File 2 — The original results of the above study (non-essential files are deleted to keep the file size under the limit set by BMC bioinformatics). [file 1471-2105-5-147-S2.bz2 › chilibotAdditionalFile2/dip05/69ID2966143E269/html/RECB_GAM.html]

 


 **RECB** and **GAM** 
  
Found 22 abstracts in PubMed, retrieved 05.  
 

 What does Google say? 
 PDF only 
| .edu only 

---

**Interactive relationship** (e.g. stimulation, inhibition, etc)

**Inhibitory relationship**- It may be inferred that lambda  **Gam**  protein, which otherwise inhibits RecBCD DNase and thus permits the growth of this phage, is bound by the helicase domain of  **RecB** .  Ref: 11130867 Res Microbiol, 2000
**Neutral relationship**
